# Magnitude of Vancomycin-Resistant *Enterococci* (VRE) Colonization among HIV-Infected Patients Attending ART Clinic in West Amhara Government Hospitals

**DOI:** 10.1155/2018/7510157

**Published:** 2018-12-31

**Authors:** Manamenot Agegne, Bayeh Abera, Awoke Derbie, Gizachew Yismaw, Melashu Balew Shiferaw

**Affiliations:** ^1^Amhara Public Health Institute (APHI), Bahir Dar, Ethiopia; ^2^Department of Medical Microbiology, Immunology and Parasitology, College of Medicine and Health Sciences, Bahir Dar University, Bahir Dar, Ethiopia; ^3^Center for Innovative Drug Development and Therapeutic Trials for Africa (CDC-Africa), Addis Ababa University, Addis Ababa, Ethiopia

## Abstract

**Background:**

*Enterococci* that colonize the intestinal tract of immunocompromised patients are an important cause of nosocomial infections. Data on the prevalence of vancomycin-resistant *Enterococci* (VRE) and its antimicrobial susceptibility patterns and associated factors are scarce in the present study area. Therefore, this study was conducted aimed at determining the prevalence of VRE colonization among HIV-infected patients attending ART clinic at West Amhara Government Hospitals.

**Methods:**

A cross-sectional study was conducted from 1 February 2017 to 31 May 2017. A total of 349 HIV patients were included in the study. A pretested structured questionnaire was used to collect sociodemographic data and possible associated factors for VRE colonization. Identification and confirmation of *Enterococci* from stool sample was performed based on the standard procedures. Antimicrobial susceptibility testing was done using the Kirby–Bauer disk diffusion method on the Muller–Hinton agar plate as per the standard protocol, and resistance profile of the isolates was determined according to Clinical and Laboratory Standards Institute (CLIS). Data were analyzed using SPSS v23. Descriptive analysis was used to visualize differences within data. Moreover, the stepwise logistic regression model was done to assess factors associated with VRE colonization. *P* value was set at 0.05 to indicate statistical significance difference.

**Results:**

The overall colonization status of *Enterococci* was at 63% (220/349). The VRE colonization was at 17 (7.7% (95% CI: 4.9–12.0)). Among *Enterococcal* isolates tested for antimicrobial susceptibility, 142 (64.5%) were found resistant to two or more antibiotics. Antibiotic treatment (for >2 weeks) and history of hospital admission in the last six month were found statistically associated for VRE colonization (AOR = 10.18, (95% CI: 1.9–53.20)) and (AOR = 20.17; (95% CI: 5.22–77.93)), respectively.

**Conclusions:**

The observed VRE with multidrug resistance colonization need a periodic surveillance of antimicrobial testing to detect emerging resistance and prevent the spread of further drug resistance.

## 1. Background

Nosocomial infections with *Enterococci* are a major concern at many hospitals and have been rapidly increasing in many countries worldwide [1]. *Enterococci* are well known antibiotic-resistant opportunistic pathogens commonly recovered from patients who received multiple courses of antibiotics and hospitalized for prolonged periods [2]. They are the second more frequently reported cause of surgical wound infection and nosocomial urinary tract infection (UTI) and the third most frequently reported cause of bacteremia [1, 3]. Especially, *Enterococcus faecalis* and *Enterococcus faecium* have become causes of international concern from the genus [4].

Vancomycin-resistant *enterococci* (VRE) belong to the most important nosocomial pathogens worldwide, and they usually cause infections in severely debilitated, immunocompromised patients, like HIV-infected patients, who undergo prolong intensive antibiotic therapy [1]. The acquisition of VRE has seriously affected the treatment and infection control of these organisms. VRE particularly *E. faecium* strains are frequently resistant to all antibiotics that are effective treatment for vancomycin-susceptible *Enterococci*, which leave clinicians treating VRE infections with limited therapeutic options [5].

Various risk factors for VRE colonization were reported before, including previous exposure to multiple antibiotics [6], presence of previous hospitalization, and prolonged duration of vancomycin use (≥7 days) [7]; catheterizations; comorbid conditions such as renal failure; underlying diseases like diabetes; and presence of surgical procedure [2, 8].

Due to high level of resistance to multiple antimicrobial agents, *Enterococcus* becomes important in health facility-based settings. HIV-infected patients who were repeatedly exposed to the healthcare environment had higher VRE colonization than other groups [9]. Data regarding the magnitude of VRE and its associated factors have been released at different health facilities; however, these data are missing in our study site. Therefore, this study was conducted to determine the prevalence of VRE colonization among HIV-positive patients attending ART clinic at West Amhara Government Hospitals.

## 2. Methods and Materials

### 2.1. Study Design, Setting, and Period

A cross-sectional study was conducted among HIV-infected patients visiting randomly the selected three government hospitals in *Amhara* regional State (Debre Markos Referral Hospital (DMRH), Debre Tabor General Hospital (DGH), and Felege hiwot Referral Hospital (FHRH)) from 1 February 2017 to 31 May 2017. The hospitals, located in the northwest part of Ethiopia, were providing different healthcare services, like emergency, inpatient, and outpatient services for about 12 million people.

### 2.2. Sample Size, Sampling Method, and Population

Sample size was determined using Epi info version 3.5.1 considering 95% confidence level, 5% marginal error, and VRE prevalence of at 7.8% [10], making the total sample size 349. Almost an equal number of participants were taken from each hospital for convenience even though the hospitals had different number of HIV patients in their respective ART clinics: from DGH (*n*=114), DMRH (*n*=117), and FHRH (*n*=118). Systematic random sampling technique was used to select study participants. Our sampling frame was the appointment log book of ART patients. Each participant was selected using a random number table based on their appearance from the appointment log book. Those HIV patients under antibiotics treatment within two weeks at the time of data collection were excluded from the study as stated previously [11].

### 2.3. Data Collection

Sociodemographic data and related clinical data suggested to be connected with VRE colonization were collected using the pretested interview-based structured questionnaire.

#### 2.3.1. Stool Sample Collection and Transportation

Patients were instructed and provided with wide-mouthed, sterile plastic containers to bring about 5–10 g stool specimens. The collected stool specimen from each hospital was transported to Amhara Public Health Institute (APHI) using Carry Blair Transport Medium (OXOID LTD, UK). Immediately after collection, approximately one gram of the fecal specimen was placed on a medium and stored in 2–8°C, until transported to the laboratory for processing.

#### 2.3.2. Bacterial Isolation and Identification

Stool samples were streaked on Bile Esculin Azide Agar (BEAA) (Hardy Diagnostics, Santa Maria, USA) and incubated for 24 hours at 37°C. Plates were observed for appearance of characteristic colonies with dark halo center. Characteristic colonies were selected randomly for characterization and identified presumptively as *Enterococci* [12] by the following phenotypic tests: (a) Gram stains; only plates that yield Gram-positive cocci in pairs or short chains were studied further. (b) Catalase test; catalase test was performed on suspected colonies according to standard microbiological procedure [12] and only microbial growth which yield negative result for catalase production were considered further. (c) Growth in 6.5% NaCl; similar colonies from each plate were picked and inoculated in to brain heart infusion (BHI) broth (OXOID LTD, UK) containing 6.5% NaCl and incubated at 37°C for 24–48 hours, and growth in the medium were indicated by turbidity [13]. (d) Growth at 45°C; colonies were picked, inoculated into BHI broth, and incubated at 45°C for 24 hours, and growth in the medium were indicated by turbidity. An isolate fulfilling the above criteria were considered as an *Enterococcus* species [12]. For further identification, stock cultures were stored at BHI broth containing 50% glycerol (Biochemica Synthesis Service, Germany) at −20°C.

#### 2.3.3. Antimicrobial Susceptibility Testing

Antimicrobial susceptibility testing for each isolate was performed using the Kirby–Bauer disk diffusion method on Muller–Hinton agar (OXOID, UK). From pure culture, selected colonies of bacteria were taken and transferred to a tube containing sterile nutrient broth (OXOID, UK) and incubated at 37°C until the turbidity of the suspension becomes adjusted to a McFarland standard (Hardy diagnostics, Canada) 0.5. Then, the suspension was spread evenly on Muller–Hinton agar based on Clinical and Laboratory Standards Institute (CLIS). The medium after exposed to a concentration gradient of antibiotic discs were incubated at 37°C for 18–24 hours. Grades of susceptibility pattern were recognized as sensitive and resistant by comparison of zone of inhibition according to the CLSI guidelines. Antimicrobial susceptibility patterns of *Enterococci* were assessed against the following antibiotic discs: vancomycin (30 *μ*g), ampicillin (10 *μ*g), nitrofurantoin (300 *μ*g), tetracycline (30 *μ*g), ciprofloxacin (5 *μ*g), chloramphenicol (30 *μ*g), erythromycin (15 *μ*g), norfloxacin (30 *μ*g), and doxycycline (30 *μ*g) (all from Abtek Biologicals Ltd.).

### 2.4. Quality Control

The reliability of the study findings was guaranteed by implementing the standard quality measures through the whole process of the laboratory work and data collection. All culture media were prepared following the manufacturer's instruction. The batch of prepared media was checked for sterility by incubating the plate at 37°C for 24 hrs. Moreover, *E. faecalis* ATCC 29212 and *Staphylococcus aureus* ATCC 25923 standard strains were used as a quality control [14].

### 2.5. Data Analysis

Collected data were entered, cleared, and analyzed using the SPSS statistical software package, Version 23.0 (IBM Corp. Released 2011. IBM SPSS Statistics for Windows. Armonk, NY: IBM Corp.). Descriptive analysis such as proportion, median, and variance were used to visualize differences within data. Moreover, stepwise logistic regression model was used to assess factors associated with VRE colonization in terms of the odds ratio and its 95% confidence interval (CI). *P* value was set at 0.05 to indicate statistical significance difference.

## 3. Ethical Approval

Ethical clearance was obtained from Bihar Dar University college of Medicine and health ethical review board (IRB). Official permission and an informed written consent were obtained from the hospitals and each study participant, respectively. The consent of children was obtained from their family or guardian. The confidentiality of the information was maintained. The VRE positive results were communicated to their physicians for appropriate management.

## 4. Results

### 4.1. Sociodemographic Characteristics

A total of 349 study participants were included in this study, of which, 202 (57.9%), were females. The mean age of the patients was at 36.4 years with standard deviation of 12.9 years. Majority of the study participants, 268 (76.8%), came from urban settings. Less than half of the participants were illiterate (133 [38.1%]) and married (151 [43.3%]) (Table 1).

### 4.2. Prevalence of VRE Colonization

Among the 349 study participants, *Enterococci* were isolated from 220 (63%), of which, 67 (30.5%) were from FHRH, 71 (32.3%) from DGH, and 82 (37.3%) from DMRH. In turn, amongst the total 220 isolates of *Enterococci*, 17/220 (7.7% (95% CI: 4.9–12.0)) were VRE. Proportion of VRE from FHRH, DGH, and DMRH was 4 (6.0%), 7 (9.9%), and 6 (7.3%), respectively.

### 4.3. Antimicrobial Resistance Profile of *Enterococci*

Of the total 220 *Enterococcal* isolates tested against the commonly available antimicrobial agents, 142 (64.5%) were found apparently resistant to two or more antimicrobials. Specifically, 94 (42.7%) were found resistant to erythromycin. Moreover, 83 (37.7%) and 68 (30.9%) *Enterococcal* isolates were reported to be resistant to tetracycline and doxycycline, respectively (Tables 2 and 3).

### 4.4. Factors Associated with VRE

All sociodemographic characteristics of the study participants such as sex, age, educational status, residence address, and marital status were not found associated with VRE colonization (*p* value > 0.05). However, most of VRE were isolated from female participants, patients aged >25 years, and HIV patients who came from urban setting (Table 4). In the multiple logistic regression model, patients with history of hospital admission in the last six month and patients having history of previous administration of antibiotics >2 weeks had >20 (AOR = 20.17; (95% CI: 5.22–77.93)) times and >10 (AOR = 10.18, (95% CI: 1.9–53.20)) times more likely to be colonized with VRE as compared with their counterparts. However, the remaining variables didn't show statistical significance difference (*p* value > 0.05) (Table 5).

## 5. Discussion

The rapid emergence of drug resistant *Enterococci* and the increasing incidence of colonization with VRE have become healthcare issues that have caused serious concern to physicians and health authorities [15]. In the present study, we included study participants from three government hospitals, which cover different geographic areas. The overall prevalence of *Enterococci* colonization in this study was at 63%, which is higher than previous reports from different hospitals in Ethiopia such as in Jimma (0.59%) [16], Felege Hiwot (0.64%) [17] and University of Gondar Teaching hospital (2.13%) [18]. Another study also reported higher *Enterococcal* isolates of 125 (78.12%) *Enterococcus faecalis* and 35 (21.88%) *Enterococcus faecium* [19]. The difference might be due to the use of *Enterococci* selective media in the present study which might increase the chance of isolation. On top of this all of our study participants were HIV positive patients in which *Enterococci* colonization is much likely due to their compromised immune system. Moreover, the gradual increase of *Enterococci* infection might have contributed to the increased prevalence as evidenced by other studies [20]. However, our finding was lower than reports from elsewhere in the World like, Egypt at (82.83%) [21] and Hijazii in hospitalized patients at (94%) [22].

The overall prevalence of VRE in this study was 7.7%, which is in line with reports from government medical collage Seurat (8%) [15]. However, it was lower than from a report in Taiwan (11.3%) [23], southern Iran (22%) [24] and Shiraz (14.7%) [25]. The lower prevalence in the present study might be due to the variation in the study participants and the methods employed for detection.

On the other hand, the prevalence VRE in the present study was higher than a report from Konkuk University School of Medicine, Seoul (4.5%) [22] and Iran (4.38%) [26]. This deference might be attributed by the variation in the study population where most of the study participants in the present study had habit of animal contact and this was supported by Bekele and Ashenafi that reported 100% VRE from faces of chicken and cattle in Ethiopia [27].

The *Enterococci* isolates in the present study were found variably resistant against the tested antibiotics such as ampicillin at (20.9%) erythromycin (42.7%) doxycycline (27.7%), tetracycline (28.6%), ciprofloxacin (37.7%) and chloramphenicol at (30.9%). The resistance profile reported in the present study was higher than a previous study in Gondar [18]. This variation might be due to the fact that we are in the time of gradual change in the antibiotic resistance pattern of isolates. However, the antimicrobial resistance profile observed in this study was found less than a previous study done in Iraq against ampicillin (71.4%), ciprofloxacilin (71.5%) [28] and erythromycin (73.12%) [19]. In the later study, the participants were all hospitalized patients who were taking different antibiotics that might be contributed for emergence of high rate of drug resistant microorganisms including *Enterococci* compared with other patients.

Moreover, our study showed that all VRE isolates were resistant to ampicillin and were multidrug resistant (MDR). This result was in line with the previous finding in Gondar, Ethiopia [10]. In contrast, the *Enterococci* isolates of this study showed relatively better sensitivity for nitrofurantoin than to vancomycin, norfloxacin, ampicillin, ciprofloxacin, and chloramphenicol. This finding was also supported by a previous report in Egypt [21].

With regard to the associated factors assessed for VRE colonization, patients with history of hospital admission in the last six months had about 20 times more likely the chance of getting VRE colonization as compared with those with no recent history of hospital admission in the last six months (*P* value < 0.001). This result was comparable with reports from Spain [29]. This study also showed that prevalence of VRE colonization with patients having history of previous administration of antibiotics greater than two weeks had 10 times more likely had the chance of getting VRE colonization as compared with those with no history of previous administration of any antibiotics (*P* value < 0.012). Similar reports from Gondar, Ethiopia, showed that antibiotic exposure can cause the emergence of VRE and creates for the survival of *Enterococci* in hospital environment due to their intrinsic resistance to several commonly used antibiotics and their ability to acquire resistance genes [10]. The rest variables tested in the model did not show statistical significant difference (*P* value > 0.05). Of course, similar finding was reported in the US [9].

Due to resource constraints, it was not possible to determine the different species of *Enterococci* which might all have a determinant factor to show the complete picture of the isolates in the studied area.

## 6. Conclusions

The emergence of VRE linked with multidrug resistance was observed in this study. Hence, a periodic surveillance on antibiogram of VRE in hospitals is essential to contain the spread of antimicrobial resistance. Efforts should also be made to prevent VRE colonization of patients with immunesuppression.

## Figures and Tables

**Table 1:** Sociodemographic characteristics of study participants (*n*=349) at the West Amhara Government Hospitals, May, 2017.

Sociodemographic characteristics	Frequency (%)
*Sex*	
Female	147 (42.1)
Male	202 (57.9)

*Age in years*	
<9	8 (2.3)
10–14	18 (5.2)
15–24	34 (9.7)
25–59	275 (78.8)
60+	14 (4.0)

*Residence addresses*	
Urban	268 (76.8)
Rural	81 (23.2)

*Educational status*	
Illiterate	133 (38.1)
Primary school	120 (34.4)
Secondary school	57 (16.3)
Above high school	39 (11.2)

*Marital status*	
Single	87 (24.9)
Married	151 (43.3)
Divorced	79 (22.6)
Widowed	32 (9.2)

*Total*	349 (100)

**Table 2:** Antimicrobial resistance profile of *Enterococci* among ART patients at West Amhara Government Hospitals, May, 2017 (*n*=220).

Antimicrobial agents	Susceptible, *N* (%)	Resistant, *N* (%)
Vancomycin	203 (92.3)	17 (7.7)
Ampicillin	174 (79.1)	46 (20.9)
Nitrofurantoin	214 (97.3)	6 (2.7)
Chloramphenicol	152 (69.1)	68 (30.9)
Tetracycline	157 (71.4)	63 (28.6)
Doxycycline	159 (72.3)	61 (27.7)
Erythromycin	126 (57.3)	94 (42.7)
Norfloxacin	184 (83.6)	36 (16.4)
Ciprofloxacin	137 (62.3)	83 (37.7)

**Table 3:** Profile of multidrug-resistant pattern of VRE (*n*=17) among ART patients in West Amhara Government Hospitals, May, 2017.

Resistance rate	Combination of antibiotics	Number of isolates tested
R3	VAN, AMP, CHL	2
R3	VAN, AMP, ERY	2
R3	VAN, AMP, NOR	1
R4	VAN, AMP, TET, DOC	1
R5	VAN, AMP, TET, ERY, DOC	1
R5	VAN, AMP, CIP, CHL, ERY	1
R5	VAN, AMP, CIP, ERY, NOR	1
R6	VAN, AMP, TET, CIP, NOR, DOC	1
R6	VAN, AMP, TET, CIP, ERY, DOC	3
R6	VAN, AMP, NIT, CIP, CHL, ERY	1
R6	VAN, AMP, TET, CIP, NOR, DOC	1
R7	VAN, AMP, NIT, TET, CIP, CHL, DOC	1
R8	VAN, AMP, TET, CIP, CHL, ERY, NOR, DOC	1
Total		17

ERY, erythromycin; TET, tetracycline; AMP, ampicillin; VAN, vancomycin; DOC, doxycycline; NIT, nitrofurantoin; CIP, ciprofloxacin; NOR, norfloxacin; CHL, chloramphenicol; *R*, resistance. *R*3–*R*8 = number of antibiotic-resistant isolates from 3 to 8, respectively. MDR = organisms resistant to ≥2 antibiotics.

**Table 4:** Sociodemographic characteristics and prevalence of VRE among ART patients in West Amhara Government Hospitals, May, 2017.

Demographic characteristics	Vancomycin		*P* value
	Sensitive	Resistant	
*Sex*			
Male	89	5	0.254
Female	114	12	

*Age group*			
<9	5	0	0.997
10–14	9	0	
15–24	19	2	
25–59	162	14	
60+	8	1	

*Residence address*			
Urban	156	14	0.604
Rural	47	3	

*Educational status*			
Illiterates	79	5	0.382
Primary school (1–8)	66	10	
Secondary school (9–12)	35	2	
Above high school	23	0	

*Marital status*			
Single	52	3	0.804
Married	85	9	
Divorced	48	4	
Widowed	18	1	

**Table 5:** Multiple logistic regression analysis of prevalence of VRE colonization at West Amhara Government Hospitals, May, 2017.

Risk factor	Category	Vancomycin		COR (95% CI)	AOR (95% CI)	*P* value
		Sensitive	Resistant			
Pervious antibiotic treatment (>2 weeks)	No	97	2	1		0.012
	Yes	106	15	6.86 (1.53–30.79)	10.18 (1.95–53.20)	

History of hospital admission in the last 6 months	No	193	10	1		0.001
	Yes	10	7	13.51 (4.25–42.94)	20.17 (5.22–77.93)	

HAART duration	<2 years	11	3	1		0.92
	>2 years	192	14	3.74 (0.934–14.97)	—	

Renal failure	No	196	15	1		0.319
	Yes	7	2	3.73 (0.71–19.57)	—	

## Data Availability

All data generated during this study are included in this manuscript.

## Abbreviations

APHI: Amhara public Health Institute
ART: Antiretroviral therapy
ATCC: American Type Culture Collection
BEAA: Bile Esculin Azide Agar
BHI: Brain heart infusion
CLSI: Clinical Laboratory Standards Institute
DGH: Debretabor General Hospital
DMRH: Debre Markos Referral Hospital
FHRH: Felege Hiwot Referral Hospital
VRE: Vancomycin-resistant *enterococci*.
